# Synthesis, Curing Behaviors and Properties of a Bio-Based Trifunctional Epoxy Silicone Modified Epoxy Thermosets

**DOI:** 10.3390/polym14204391

**Published:** 2022-10-18

**Authors:** Dan Qian, Jiahai Zhou, Jieyuan Zheng, Jun Cao, Jintao Wan, Hong Fan

**Affiliations:** 1 State Key Laboratory of Chemical Engineering, College of Chemical and Biological Engineering, Zhejiang University, Hangzhou 310027, China; 2 Zhejiang Chuanhua Chemical Group Co., Ltd., Hangzhou 311200, China; 3 MOE Engineering Research Center of Historical and Cultural Heritage Protection, School of Materials Science and Engineering, Shaanxi Normal University, Xi’an 710062, China

**Keywords:** eugenol, silicone, epoxy resin, toughness, thermal stability

## Abstract

Tremendous effort has been focused on improving the toughness of epoxy, but the common approaches diminish the mechanical properties. In this work, a new silicone-modified trifunctional epoxy monomer SITEUP is synthesized from the hydrosilylation transformation of eugenol epoxy (EPEU) and tris-(dimethylsiloxy)phenylsilane. The chemical structures and curing kinetics of SITEUP are investigated based on ^1^H-NMR, ^13^C-NMR, MADLI-TOF-MS, and DSC analyses. SITEUP is introduced into DGEBA/IPDA systems as a functional modifier in varied loadings for toughening the resulting epoxy thermosets. The impact strength of the modified epoxy thermosets containing 20% SITEUP is 84% higher than that of the pristine epoxy thermoset and also maintains high flexural strength. Further morphology study reveals that the plastic deformation caused by siloxane segments is the key factor accounting for the enhanced toughness of the finalized epoxy thermosets. Si-O-Si segments incorporated into the thermosetting network could absorb more energy by increasing the mobility of polymer chains under external stress and led to improved thermal stability and damping characteristics. In addition, SITEUP is able to decrease the surface tension and increase the hydrophobic properties of the resultant epoxy materials.

## 1. Introduction

Epoxy resin is a kind of synthetic polymer that is widely used in coatings, electronic packaging, civil engineering and other industries due to its excellent adhesive property, mechanical stiffness, chemical stability and electrical insulating property [1,2,3,4]. However, the major drawback of cured epoxy thermosets is the brittleness caused by their highly crosslinked network structures, which limit their applications in many fields [5,6,7,8,9,10]. Many researchers incorporate nanofillers [11,12], liquid rubber [13,14] and thermoplastic polymers [15] to improve the toughness of cured epoxy resins, but this always decreases some other mechanical properties and processabilities.

Silicone refers to a class of compounds with siloxane segments and other organic substituents. The siloxane chain exhibits excellent flexibility, and the Si-O bond with high bond energy shows superior thermal stability. Moreover, the reactive functional groups of silicone perform good reactivity and compatibility with other polymers [16,17,18,19]. Two commonly used methods are used for modifying epoxy resin with silicone: physical and chemical modification. The physical modification involves a simple physical mixing process by blending silicone and epoxy resin to form a system with excellent comprehensive performance [20]. Chemical modification depends on the chemical reaction between the reactive group (such as amino, hydroxyl, epoxy groups and others) attached to silicones and the reactive group of epoxy prepolymers or curing agents to form modified thermosetting epoxy networks [21,22]. Lee et al. [17] grafted aminopropyl to polydimethylsiloxane used to react with a tetrafunctional epoxy prepolymer. The results showed that dispersed silicone rubbers effectively reduce the internal stress of the tetrafunctional epoxy thermosets by reducing the flexural modulus and the coefficient of thermal expansion. Ahmad et al. [18] synthesized a kind of silicone-modified epoxy resin via a ring-opening polymerization reaction between diglycidyl ether of bisphenol A (DGEBA) and hydroxyl-terminated poly(dimethyl siloxane) (HPDMS). The resulting siloxane-modified epoxy-polyamide coatings exhibited superior physicomechanical, thermal resistance and anticorrosive performance. However, the common silicone resins result in poor compatibility with epoxy matrices, which will lead to phase separation and a significant decrease in mechanical properties.

With the increasing environmental concern and the limited resources associated with petroleum, biomass materials with similar structures have been investigated in recent years. Eugenol is a renewable and less toxic compound with a special structure that can be obtained from natural extracts [23,24]. The phenolic hydroxyl group on eugenol can react with epichlorohydrin to form an epoxy group, and the ally group can be linked via a hydrosilylation reaction [25,26]. Many researchers have synthesized a great variety of epoxy resins from eugenol. Chen et al. [27] presented a novel approach to synthesizing a 100% bio-based epoxy thermoset with eugenol, including the preparation of four fully bio-based epoxy compounds and the self-curing reaction of these monomers. Faye et al. [28] prepared a new epoxy monomer tri(epoxized-eugenyl)phosphate (TEEP) derived from eugenol and cured with two different amines. These new bio-based thermosets exhibited good thermomechanical properties and excellent thermal properties with high char yields. Wan et al. [29] used eugenol, cyanuric chloride and *m*-CPBA to synthesize a eugenol-based epoxy monomer (TEU-EP), which was well cured by 3,3′-diaminodiphenyl sulfone (33DDS). TEU-EP/33DDS had higher Young’s modulus, hardness and glass transition temperature compared with DGEBA/33DDS. Li et al. [30] developed a series of silicone-bridged difunctional epoxy monomers derived from eugenol. After being cured by DDS, the obtained material exhibited a dielectric permittivity as low as 2.8 and good intrinsic flame retardancy with an LOI value higher than 31, far outperforming DGEBA counterpart.

**Scheme 1:**
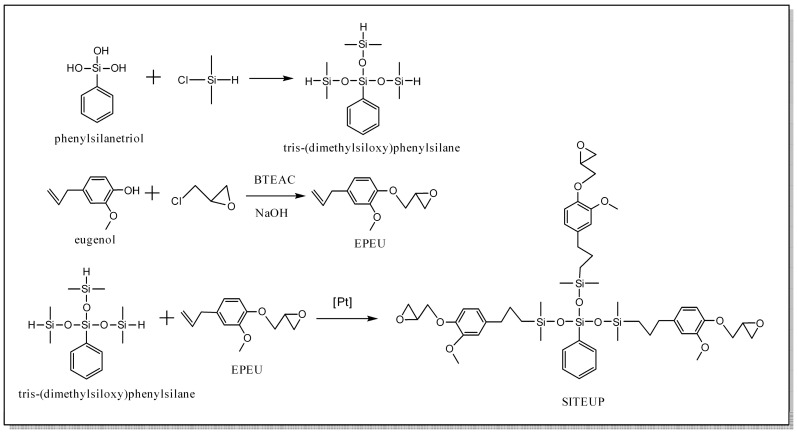
Synthesis of trifunctional epoxy silicone monomer.

In this research, a new eugenol-based epoxidized silicone resin (SITEUP) was synthesized (Scheme 1) and then cured with epoxy resin to improve toughness. The mechanical properties, thermal properties and hydrophobicity of epoxy thermosets were investigated and the toughening mechanisms of SITEUP were proposed. The synthesis, characterization and ultimate properties are discussed in detail.

## 2. Materials and Methods

### 2.1. Materials

Phenylsilanetriol was synthesized in our lab. Toluene, pyridine, epichlorohydrin, sodiumhydroxide and benzyl triethylammonium chloride were purchased from China Reagent Co. Dimethylchlorosilane and eugenol were purchased from Energy Chemical. Platinum (0)-1,3-divinyl-1,1,3,3-tetramethyl disiloxane complex solution (Karstedt catalyst, 2 wt% Pt xylene solution), diglycidyl ether of bisphenol A (DGEBA) and isophorone diamine (IPDA) were used as received.

### 2.2. Synthesis of SITEUP

#### 2.2.1. Synthesis of Tris-(dimethylsiloxy)phenylsilane

An amount of 5.00 g of phenylsilanetriol, 15.9 g of dimethylchlorosilane, 100 mL of toluene and 11.6 g pyridine were mixed in a three-necked round bottom flask with stirring and refluxing for 7 h. The organic filtrate obtained by filtration was washed with deionized water 3~5 times and dried with anhydrous magnesium sulfate. Then, the solvent was removed by rotary distillation and the product was obtained by further vacuum distillation.

#### 2.2.2. Synthesis of EPEU

Epoxidation of eugenol was carried out by reacting 350 g of epichlorohydrin, 100 g of eugenol and 3.5 g benzyltriethylammonium chloride into a flask under 110 °C for 2 h. Then, 135 g of 20% NaOH(aq) was added dropwise into the mixture in 5 h and the reaction continued for 5 h. The crude product was purified by washing with deionized water and then the unreacted epichlorohydrin was removed by distillation under reduced pressure. The obtained cured product was recrystallized twice at low temperatures in methanol to afford EPEU crystals.

#### 2.2.3. Synthesis of SITEUP

An amount of 100 g of EPEU, 200 mL toluene and 0.2 g of Karstedt catalyst (Pt 2 wt%) were mixed into a 500 mL round bottom flask in nitrogen protection under 70 °C for 1 h, followed by adding 60 g of tris-(dimethylsiloxy)phenylsilane dropwise and holding for another 12 h to finish the hydrosilylation. The flask was cooled to room temperature and the catalyst was removed by silica gel. Finally, SITEUP was obtained after the removal of toluene and unreacted EPEU by vacuum distillation.

### 2.3. Preparation of Cured Epoxy Resin

The DGEBA and SITEUP were mixed well and then degassed at 90 °C for 2 h. Then IPDA was added into the mixture at a stoichiometric ratio (mole ratio of amine hydrogen to epoxy group = 1:1), stirred homogenously and defamed for 5 min in a vacuum. The obtained reaction mixture was poured into the mold and cured at 80 °C for 2 h and then 150 °C for 2 h. After cooling to room temperature, demolding gives to the cured epoxy thermoset, which was used for the property studies.

### 2.4. Measurements

The ^1^H-NMR and ^13^C-NMR analyses were performed on a Bruker Avance 400 NMR spectrometer. CDCl_3_ was used as solvent and tetramethylsilane (TMS) as the internal standard.

The Matrix-Assisted Laser Desorption/Ionization Time of Flight Mass Spectrometry analysis was performed on a Bruker MALDI-TOF-MS spectrometer. Tetrahydrofuran (THF) was selected as the solvent and 2, 5-Dihydroxybenzoic acid (DHB) as the matrix.

The glass transition temperature (*T*_g_) of the cured sample was measured on a differential scanning calorimeter (DSC Q200, TA Instruments). The heating rate was 10 °C/min from 50 to 200 °C and the test was under a nitrogen atmosphere (20 mL/min). This DSC was used to examine the curing process of the epoxy systems. The SITEUP and IPDA were quickly mixed at a stoichiometric ratio (molar ratio of amine hydrogen to epoxy group = 1:1). Then, the samples (8–10 mg) were sealed in aluminum pans to run DSC scans.

The bending strength of the cured sample (120 × 10 × 4 mm^3^) was measured according to GB/T 9341–2000 with a universal testing machine (Zwick/Roell Z020). The crosshead speed was 1.0 mm/min.

The non-notched impact strength of the cured sample (120 × 15 × 10 mm^3^) was measured by a Pendulum impact meter (CEAST) according to GB/T1043.1–2008.

The fracture surface of the gold-coated cured sample was examined with a Carl Zeiss Utral55 scanning electron microscopy (SEM). The scanning voltage was set at 3 KV and the magnification was 1000 times.

The thermal decomposition of the cured thermosets was analyzed on thermogravimetric analysis (TGA Q500). The sample (~3 mg) was scanned from room temperature to 800 °C (10 °C/min) under nitrogen flow (40 mL/min).

The static contact angles of the cured samples were examined with a video-based contact angle measuring device (DSA30). Contact angle values were determined at room temperature by Attention Theta.

## 3. Results and Discussion

### 3.1. Molecular Structure Characterization

**Figure 1:**
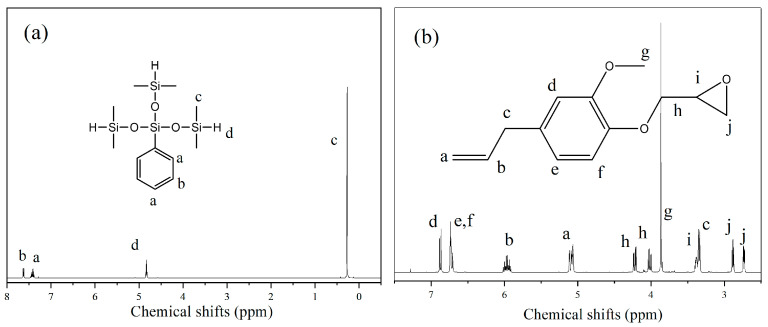
(**a**) ^1^H-NMR spectra of (**a**) tris-(dimethylsiloxy)phenylsilane and (**b**) EPEU.

**Figure 2:**
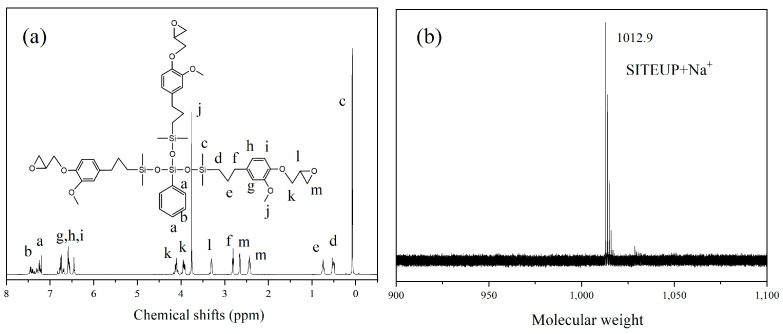
(**a**) ^1^H-NMR and (**b**) MADLI-TOF-MS spectra of SITEUP.

Triphenylsilanol and chlorodimethylsilane underwent condensation to yield tris-(dimethylsiloxy) phenylsilane and then reacted with eugenol monoepoxide via hydrosilylation. Figure 1a shows the ^1^H NMR spectrum of tris-(dimethylsiloxy) phenylsilane, from which the resonances at 7.5 ppm, 4.8 ppm, and 0.3 ppm are attributable to the aromatic ring, Si-H, and Si-CH_3_, respectively. Figure 1b shows observed signals of 2.8 and 2.7 ppm, which are denoted to the epoxy group of EPEU. The resonance around 4.0–4.2 ppm is owing to -OCH_2_-. In the subsequent transformation, the ally group of EPEU was used to react with tris-(dimethylsiloxy)phenylsilane through hydrosilylation. Figure 2a shows some significant changes appearing in the target SITEUP. In contrast to reactants, tris-(dimethylsiloxy)phenylsilane and EPEU, the signals of the allylic group (at 5.1 and 6.0 ppm) disappear while the signals of methylene appear (at 0.5 and 0.7 ppm) in SITEUP, indicating the complete hydrosilylation. The MADLI-TOF-MS spectrum of SITEUP displays the signal at 1012.9 (M + Na), which is consistent with the predicted molecular weight of the target product (1014.2). All these results support the successful achievement of SITEUP with good purity.

### 3.2. Non-Isothermal Curing Behaviors

The non-isothermal curing behaviors of SITEUP resin were studied with dynamic DSC. The fresh samples were heated at the ramp rates of 3, 4.5, 6.7 and 10 °C/min, respectively, from 25 to 250 °C under a nitrogen atmosphere (20 mL/min).

**Figure 3:**
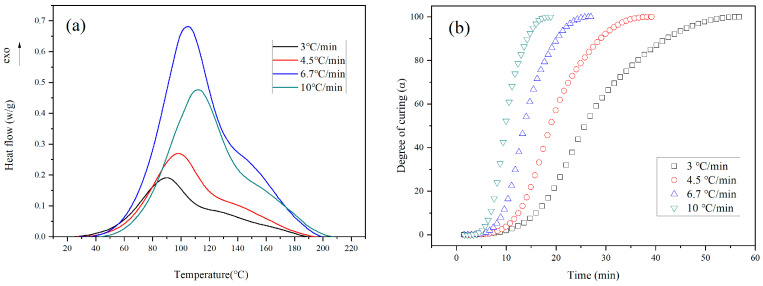
DSC heat flow and conversion graphs at multiple heating rates. (**a**) Heat flow against curing with different heating rates; (**b**) conversion as a function of time.

Figure 3a shows the non-isothermal heat flow curves of the curing reaction with different heating rates. All these DSC curves show a major exothermic peak with a minor shoulder at a high temperature. Because IPDA has two amines with varied reactivity when reacting with epoxy rings, one is an aliphatic amine and another is a cyclic aliphatic amine. The appearance of the main peak and shoulder peak corresponds to the epoxy-amine reaction involving aliphatic amine and cyclic aliphatic amine with different reactivates [31].

It is generally acknowledged that the enthalpy of the curing reaction is directly proportional to the fractional conversion, *α*, which can be calculated using Equation (1) [32,33,34,35]:

(1) $$\alpha =\frac{\Delta H_{T}}{\Delta H_{\infty }}$$

where Δ*H*_∞_ and Δ*H_T_* are the total heat release of the reaction over the whole temperature range and the heat release of the reaction at given temperature *T*.

According to the multiple heating rate DSC data, *α* values of the curing reaction at various heating rates are acquired using Equation (1). The experimental curves of *α* versus time for samples at different heating rates are compared in Figure 3b. All the conversion curves show a sigmoidal profile, which may be caused by the acceleration of the reaction intermediates [36]. Moreover, it is observed that at a higher heating rate, less reaction time is required to reach the same *α* as the heating rate increases, which means the reaction rate increases with the increase in the heating rate.

The effective activation energy of the curing reaction is obtained by conducting the model-free kinetic analysis with the Vyazovkin method [37,38,39].

(2) $$\Phi \left(E_{\alpha }\right)=\overset{n}{\underset{i=1}{\sum }}\overset{n}{\underset{j\neq i}{\sum }}\frac{I\left(E_{\alpha },T_{\alpha ,i}\right)\beta_{i}{}^{-1}}{I\left(E_{\alpha },T_{\alpha ,j}\right)\beta_{j}{}^{-1}}$$

(3) $$I\left(E_{\alpha },T_{\alpha ,i}\right)=\int_{T_{\alpha -\Delta \alpha }}^{T_{\alpha }}\exp\left[\frac{-E_{\alpha }}{RT}\right]dT$$

**Figure 4:**
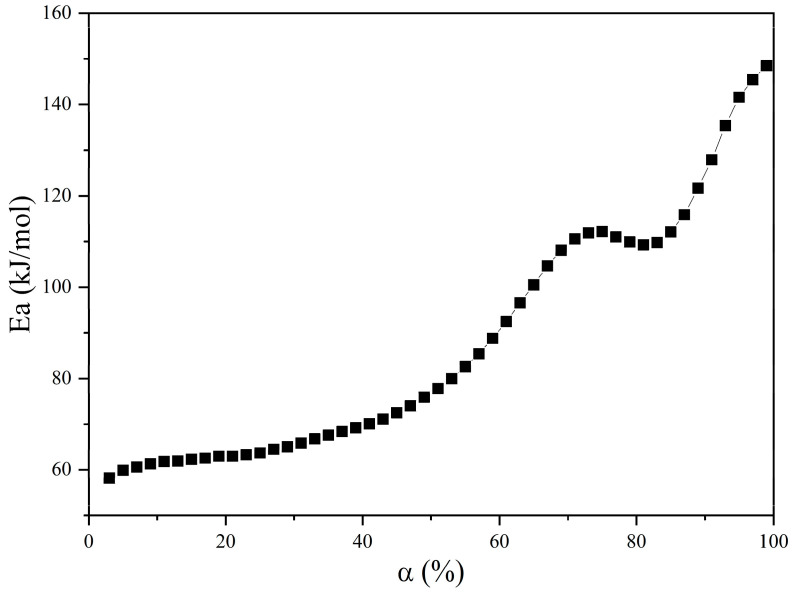
Effective activation energy as a function of conversion of SITEUP/IPDA.

The evolution of the activation energy with the degree of conversion is calculated by this isoconversional method, and the calculated values are shown in Figure 4.

There is a slight uptrend of *E*_a_ from 58 to 64 kJ/mol with conversion at the initial stage of the reaction (*α* = 1–20%). Generally, the reaction activation energy is dependent on the types of reactive species in the reaction systems and also is affected by molecular diffusion [39,40,41,42]. The initial curing reaction involves a high concentration of the reactive sites (epoxy and different types of amino groups), and the viscosity is also influential at this stage. Although the increased temperature is beneficial to reduce the viscosity, it will also lead to a rapid increased molecular weight, which in turn, will increase the viscosity and slow the molecular diffusion; therefore, the diffusion of reactive groups becomes increasingly influential, which causes the gradually increased energy barrier for the reaction. In the intermediate stage (*α* = 20–70%), the molecular weights and crosslinks increase rapidly as the curing reaction proceeds; thus, the energy barrier needs to overcome increases rapidly from ~65 to 110 kJ/mol. When 70% < *α* < 80%, the appearance of a downward trend of *E*_a_ likely indicates that cyclic amine groups of IPDA dominate the reaction. As discussed above, at a lower temperature and an initial stage of reaction, the epoxy–amine reaction mainly proceeds with aliphatic amines. With the curing process proceeding, the increased reaction temperature induces the reactivity of cyclic aliphatic amines, which increases the chance of effective collision among reactive sites. In other words, a decreasing tendency of *E*_a_ is the consequence of sharp increased active amines groups. Afterwards, *E*_a_ continues to increase until the end of the reaction, where an abrupt increase is observed. This is because an infinite molecular network begins to form, which accelerates the viscosity of the system and leads to greater steric hindrance [42]. As a result, the mobility of the molecular chain and the reactivity of the active site will decrease, which will enlarge the energy barrier quickly.

### 3.3. Glass Transition Temperature

**Figure 5:**
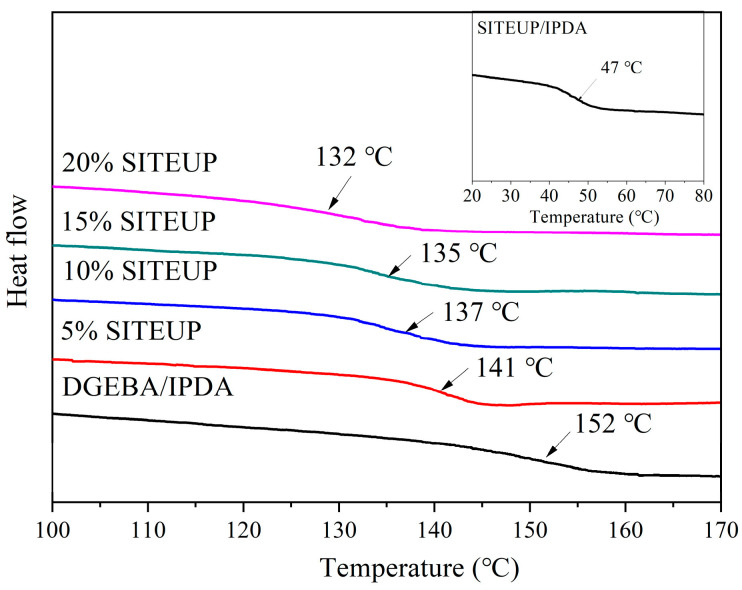
Glass transition temperatures of DGEBA/IPDA thermosets modified with different contents of SITEUP.

Figure 5 shows the glass transition temperature (*T*_g_) of epoxy thermosets with different loadings of SITEUP. The neat SITEUP thermoset exhibits the lowest *T*_g_ of 47 °C, which is attributed to the flexible siloxane chains and lowered crosslinked density, showing that SITEUP is likely more suitable for coating applications with good flexibility [43,44]. More interestingly, SITEUP can act as the reactive additive and participate in the curing process of DGEBA/IPDA, with limited decreases in *T*_g_ observed. To illustrate, with the SITEUP amount increasing from 0% to 20% and *T*_g_ decreasing from 152 to 132 °C, suggesting that the SIETUP-modified epoxy thermosets are still usable at high temperatures.

### 3.4. Dynamic Mechanical Properties

**Figure 6:**
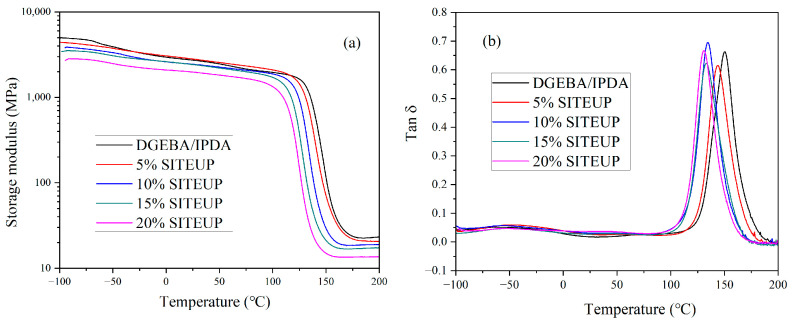
Dynamic mechanical properties of DGEBA/IPDA thermosets modified with different contents of SITEUP. (**a**) Storage modulus and (**b**) Tan δ against temperature.

Figure 6 illustrates the effect of the silicone contents on the storage modulus and the tan delta curves of the thermosets. The storage modulus displayed in Figure 6a shows that the storage modulus of epoxy resin is shifted to a lower value with the introduction of SITEUP at both the glassy state and rubber state. These changes are due to the decrease in crosslink density and the introduction of flexible segments, resulting in the increased molecular motion of the polymer chains, which is conducive to energy dissipation [44]. As a result, the addition of SITEUP improves the damping properties of cured epoxy resin.

The major relaxation process of the crosslink networks observed in Figure 6b is due to the glass transition of the epoxy thermosets [45]. With the increased contents of SITEUP in the epoxy thermoset, *T_g_* (peak relaxation temperature for tan δ) decreases from 150 °C to 131 °C. This observation is somewhat similar to the trend as compared with the DSC results. Furthermore, another minor relaxation is observed at −100 °C to 0 °C, which can be assigned to the β-transition. This minor relaxation process is owing to the localized segmental motions attributed to the hydroxypropylether sequences in the amine-cured epoxy thermosets.

### 3.5. Mechanical Properties

The flexural strength and flexural modulus of epoxy–silicone systems are listed in Table 1. With the content of SITEUP increasing from 0 to 20%, the flexural strength decreases from 94.6 to 85.3 MPa, and flexural modulus decreases from 1610 to 1280 MPa, but still could satisfy the basic requirement of the modified thermoset as a hard plastic. These results are associated with the presence of flexible siloxane linkages, free rotation of the Si-O-Si bonds and weak interface boundaries between the siloxane and epoxy thermosets [46,47].

The impact strength of epoxy–silicone systems is listed in Table 1 and the results show that the SITEUP leads to the enhanced toughness of epoxy thermosets. With 20% SITEUP loading, the impact strength of the thermoset increases from 13.9 to 25.6 kJ/m^2^—an 84% increment compared with the unmodified epoxy thermoset. The good impact toughness can be attributed to the increased energy dissipation ability of the network due to the flexible siloxane linkage introduced. The Si-O-Si skeletal bond has a longer length (1.64 Å) and larger angle (143°) than the C-C bond. As a result, these flexible siloxane linkages are able to enhance the toughness due to the higher mobility of the Si-O-Si bond, which will dissipate more impact energy [48].

**Figure 7:**
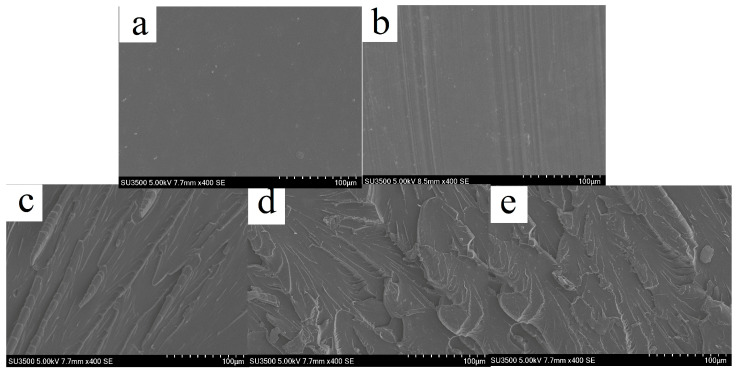
SEM images of fractures of DGEBA/IPDA thermoset with different contents of SITEUP. (**a**–**e**): 0%, 5%, 10%, 15%, 20%, respectively.

When discussing the toughening mechanisms, SEM is used to examine the impact fracture of the thermosets. In Figure 7a, the cured-DGEBA displays a smooth fracture without apparent plastic deformation, corresponding to poorer fracture toughness. After adding 5% and 10% SITEUP, river-like surfaces appear, implicating apparent crack deflection. The roughness of surfaces increases with the increasing SITEUP contents; 15% SITEUP and 20% SITEUP lead to even irregular fracture surfaces with massive, folded crimp. These morphology changes are an indication of yielding before failure and have been described as in situ toughening [49]. The improved energy dissipation and toughening are due to the formation of fibrils during the occurrence of the fracture, which results from the soft siloxane segments in the network.

### 3.6. Thermal Stability

**Figure 8:**
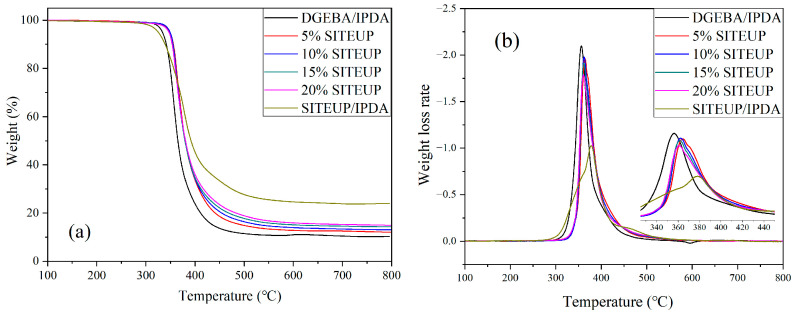
Thermogravimetric analytical curves of DGEBA/IPDA with different content of SITEUP. (**a**) TG thermographs; (**b**) DTG curves.

Figure 8 shows the TGA and DTG curves of the cured epoxy thermosets with the different content of SITEUP under a nitrogen atmosphere. The initial decomposition temperature for 5% weight lost (*T_−_*_5%_), peak temperature (*T*_max_) and solid residue at 800 °C are listed in Table 2. The thermal stability is generally improved by the introduction of SITEUP. The *T_−_*_5%_, *T*_max_ and residual yield of all the silicone-modified epoxy systems are higher than that of neat resin.

Table 2 shows that the *T_−_*_5%_ and *T*_max_ of the thermosets containing 5% SITEUP are increased by 14.9 and 8.9 °C, respectively. The *T_−_*_5%_ and *T*_max_ of the other thermosets show varied extents of improvement. The Si-O group of SITEUP needs higher thermal energy to break, and its vibration can dissipate the thermal decomposition energy [50], giving rise to the increase of *T_−_*_5%_ and *T*_max_. However, with the further increase in SITEUP, the negative effect caused by lower cross-lining density will overweigh the positive effect of the Si-O group, which decreases the thermal stability of the neat SITEUP thermoset.

The residual yield of cured samples improves linearly as the SITEUP content increases. When its content is 20%, the residual yield increases by 45.6% compared with the pure epoxy thermoset (10.3%). This is mainly the result of the formation of carbon–silicon residue. As previously reported, the decomposition of silicone leads to the formation of the silicone-containing group, which will participate in the crosslinked carbonization and eventually generate carbon–silicon [51]. The carbon–silicon residue provides a thermal insulation effect and prevents gas evolution. In other words, the presence of the SITEUP converts the usual organic decomposition to partially inorganic decomposition and achieves ultimate improvement in the flame retardation of the epoxy system [52].

### 3.7. Hydrophobic Properties

**Figure 9:**
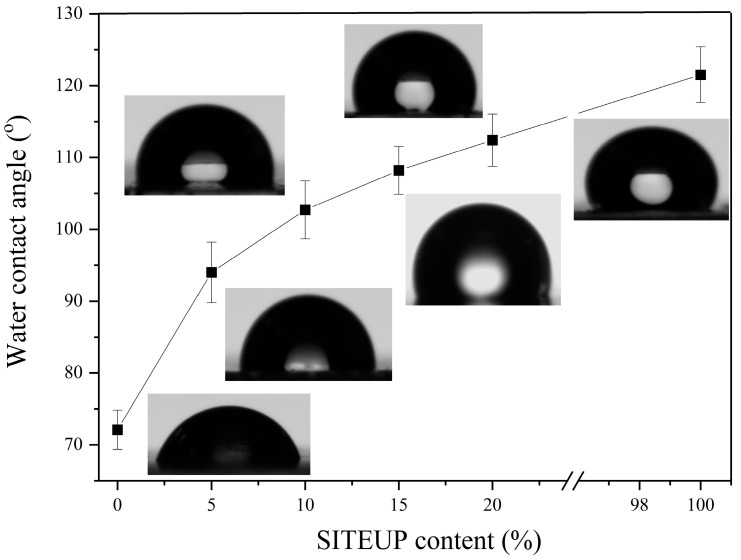
Water contact angles of DGEBA/IPDA thermosets with different content of SITEUP.

The static water contact angles of the cured samples are compared in Figure 9. Pure epoxy thermoset has a contact angle of 72°, indicating the hydrophilic surface of the material. After adding SITEUP, however, the contact angles of the modified epoxy thermoset increased significantly. When 5% SITEUP is incorporated, the contact angle of the thermosets reaches 94°, which indicates the thermoset has changed from a hydrophilic material to a hydrophobic surface; 20% of SITEUP reaches 110°. Liu et al. [53] revealed that surface tension has a close relationship with the static contact angle of a clean solid surface. The siloxane segments in the main chain of the silicones are surrounded by non-polar aliphatic groups (Si-CH_3_), contributing to the low surface tension of this material [54]. By adding SITEUP to the modified epoxy system, surface tension is decreased, and hydrophobicity of the epoxy resin is achieved; therefore, the water contact angles of the cured epoxy thermosets become increasingly greater with the increase in the silicone content.

## 4. Conclusions

A new silicone-modified material (SITEUP) derived from eugenol was successfully synthesized and characterized with the ^1^H NMR and MALDI-TOF-MS. Non-isothermal curing behaviors of the SITEUP/IPDA system were investigated, illustrating that the curing reaction took place in two stages due to two amines with different reactivity on IPDA. The flexural, impact tests and SEM analysis indicated that the introduction of SITEUP could improve the toughness of epoxy thermosets significantly. From TGA and DMA analyses, the thermostability and damping properties of the cured epoxy thermosets were enhanced with the addition of silicone. The water contact angle measurements proved that SITEUP-modified epoxy thermosets exhibited excellent hydrophobic characteristics. All the results show that SITEUP can be used as a functional modifier to gain great improvement in comprehensive performances and might broaden the applications of biobased epoxy silicones.

## Figures and Tables

**Table 1:** Mechanical properties of cured epoxy thermosets modified with different contents of SITEUP.

Formulation	Flexural Strength	Flexural Modulus	Impact Strength
	MPa	MPa	kJ/m^2^
DGEBA/IDPA	94.6 ± 3.1	1610 ± 89	13.9 ± 1.5
5% SITEUP	91.5 ± 2.9	1370 ± 91	15.9 ± 2.6
10% SITEUP	90.2 ± 3.5	1360 ± 54	18.8 ± 2.1
15% SITEUP	88.5 ± 4.2	1330 ± 41	22.2 ± 3.1
20% SITEUP	85.3 ± 3.9	1280 ± 57	25.6 ± 2.3

**Table 2:** Thermal stability parameters of the cured epoxy thermosets with different contents of SITEUP.

Sample Code	*T_−_*_5%_ (°C)	*T*_max_ (°C)	Char Yield at 800 °C (%)
DGEBA/IDPA	333.7	356.2	10.3
5% SITEUP	348.6	364.1	12.1
10% SITEUP	349.2	362.4	13.0
15% SITEUP	347.0	361.5	14.3
20% SITEUP	346.8	360.9	15.0
SITEUP/IDPA	325.4	378.1	23.9
